# Skin dose estimation for various beam modifiers and source-to-surface distances for 6MV photons

**DOI:** 10.4103/0971-6203.51935

**Published:** 2009

**Authors:** Girigesh Yadav, R. S. Yadav, Alok Kumar

**Affiliations:** Department of Physics D. A.V. (P.G.) College, Kanpur, India; 1 Department of Medical Physics, AMRI Hospitals, Kolkata, India

**Keywords:** Surface dose, Percentage skin dose, source-to-surface distance, Motorized 60° wedge, Multileaf collimator, Acrylic block Tray, Block, Field size

## Abstract

The purpose of this study was to learn the skin dose estimation for various beam modifiers at various source-to-surface distances (SSDs) for a 6 MV photon. Surface and buildup region doses were measured with an acrylic slab phantom and Markus 0.055 cc parallel plate (PP) ionization chamber. Measurements were carried out for open fields, motorized wedge fields, acrylic block tray fields ranging from 3 × 3 cm^2^ to 30 × 30 cm^2^. Twenty-five percent of the field was blocked with a cerrobend block and a Multileaf collimator (MLC). The effect of the blocks on the skin dose was measured for a 20 × 20 cm^2^ field size, at 80 cm, 100 cm and 120 cm SSD. During the use of isocentric treatments, whereby the tumor is positioned at 100 cm from the source, depending on the depth of the tumor and size of the patient, the SSD can vary from 80 cm to 100 cm. To achieve a larger field size, the SSD can also be extended up to 120 cm at times. The skin dose increased as field size increased. The skin dose for the open 10 ×10 cm^2^ field was 15.5%, 14.8% and 15.5% at 80 cm, 100 cm and 120 cm SSDs, respectively. The skin dose due to a motorized 60° wedge for the 10 × 10 cm^2^ field was 9.9%, 9.5%, and 9.5% at 80 cm, 100 cm and 120 cm SSDs. The skin dose due to acrylic block tray, of thickness 1.0 cm for a 10 × 10 cm^2^ field was 27.0%, 17.2% and 16.1% at 80, 100 and 120 cm SSD respectively. Due to the use of an acrylic block tray, the surface dose was increased for all field sizes at the above three SSDs and the percentage skin dose was more dominant at the lower SSD and larger field size. The skin dose for a 30 × 30 cm^2^ field size at 80 cm SSD was 38.3% and it was 70.4% for the open and acrylic block tray fields, respectively. The skin doses for motorized wedge fields were lower than for open fields. The effect of SSDs on the surface dose for motorized 60° wedge fields was not significant for a small field size (difference was less than 1% up to a 15 × 15 cm^2^ field size), but for a larger field (field size more than 15 × 15 cm^2^), the difference in a percentage skin dose was significant. The skin dose for the open field was more than that for the MLC blocked field and lower than that for the acrylic blocked tray field. The block was 25% of the 20 × 20 cm^2^ open field. Skin doses were increased as the SSD decreased and were dominant for larger field sizes. The surface dose was weakly dependent on the MLC block.

## Introduction

High Energy Medical Linear Accelerators are used for the treatment of cancer in radiotherapy; X-ray beams are used for deep-seated tumors. Mega voltage X-rays produce a skin sparing effect, whereby, a higher dose is deposited at the depth than at the skin tissue region.[1] Theoretically, the dose at the skin surface should be negligible, but this is never achieved because it has two components depending on secondary electrons produced by the photon interactions with any scattering materials such as air, the collimator jaw, patient's skin, etc. These components are secondary electrons generated in the patient[2,3] and contaminant electrons from the treatment head.[2]

There are two sources of contamination, one is treatment head materials[4,5] and other is treatment setup parameters.[6,7] The amount of these contaminant electrons and low-energy photons will affect the surface and buildup region dose.[8] The knowledge of how different parameters affect the surface and buildup region dose are essential for proper treatment.

The skin is divided into three layers epidermis, dermis, and subcutaneous fatty tissue.[9] The thickness of the epidermis and dermis is 0.05 - 0.15 mm and 1-2 mm, respectively, in most locations. The subcutaneous fatty tissue lies under the dermis. It is important to know the dose distribution of these layers before treatment because of possible biological complications of high skin doses in radiotherapy treatment, such as, desquamation, erythema, fibrosis, and necrosis.

The aim of our study is to measure the skin doses for different beam modifying devices at different source-to-surface distances (SSDs). Here we have selected beam modifiers like block, tray, motorized wedge, and MLC at three SSDs 80,100 and 120 cm, for 6 MV photon beams.

## Materials and Methods

Surface dose measurements were carried out for 6 MV photons, for various field sizes, with beam modifiers at different SSDs. Elekta precise linear accelerator (Elekta Oncology Systems, Crawley, UK) having 6 MV and 15 MV photons, and six-electron beams, with a multileaf collimator (MLC) and 40 pair of leaves, with each leaf projecting a 1 cm width at the isocentre. The linear accelerator (LINAC) has a step-and-shoot Intensity Modulated Radiation Therapy (IMRT) capability only with the 6 MV photon and not with the 15 MV photon. Measurements were carried out with the Markus parallel-plate ion chamber (0.055 cc measuring volume, 0.03 mm wall thickness, acrylic electrode, graphite coated, 5.3 mm in diameter, 2 mm electrode separation, and a 0.2 mm guard ring) with PTW electrometer–E (PTW, Freiburg, Germany). The chamber was embedded in an acrylic slab phantom. The outer dimension of the phantom was 300 mm × 300 mm, with 1 mm to 300 mm thickness. The use of a plane–parallel ion chamber with fixed plate separation on the surface and buildup region would perturbate the dose measured, to get the proper dose over response correction factor used for the markus chamber[10] on the surface and buildup region..

The central axis depth dose measurements were made in an acrylic slab water phantom. The Markus-type chamber was embedded in an acrylic water phantom, and 15 cm of backscatter thickness was used to ensure phantom scatter equilibrium. An acrylic-based material of 1.19 g/cm^3^ density and acrylic water phantom sheets of 1 mm thickness were placed one by one, above the chamber, and the charge was measured. An SSD of 100 cm was chosen for measurements. A polarizing potential of +300 V was reversed for all measurements because of a large polarity effect observed at the phantom air interface.[13] The percentage build-up region depth dose data (ranging from 0 to 2 cm depth) were measured for each setup. Readings at the phantom surface (depth = 0) were normalized to readings at the depth of dose maximum to obtain relative surface doses.

**Figure 1:**
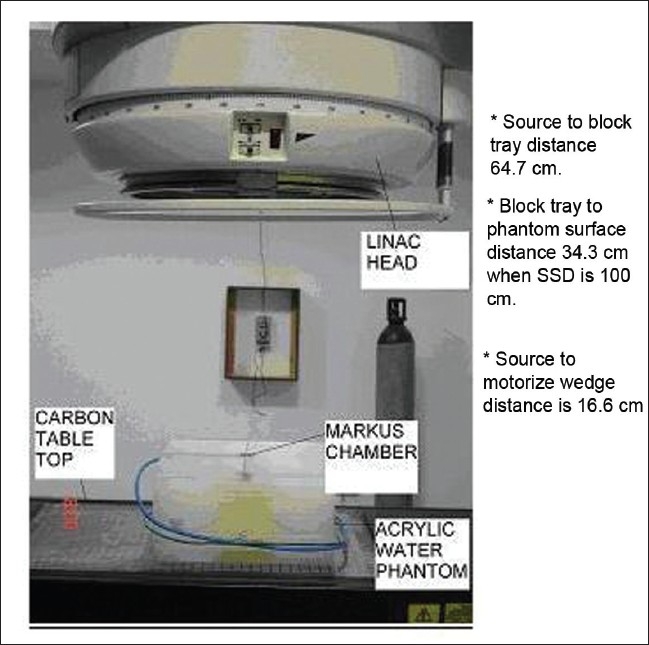
Experimental setup

**Figure 2:**
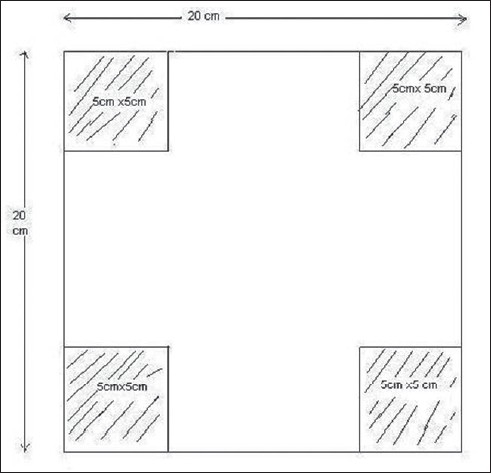
Diagram of the blocked field

Measurements of skin doses were carried out at 100 cm SSD, with different sizes of open fields, 3 × 3 cm^2^, 5 × 5 cm^2^, 10 × 10 cm^2^, 15 × 15 cm^2^, 20 × 20 cm^2^, 25 × 25 cm^2^, and 30 × 30 cm^2^ for the zero depth, and then acrylic water phantom sheets of 1 mm thickness were placed above the chamber and the charge measured for all the above-mentioned field sizes. The process was repeated up to a 2.0 cm depth. An acrylic block tray of 10 mm thickness was placed in the beam, to determine its effect on the skin dose. The tray was used to support the Cerrobend blocks and it was placed at the accessory tray holder located at 64.7 cm from the source. Surface to buildup region dose, up to a 2.0 cm depth, was measured as per the above-mentioned procedure. The effect of a motorized 60° wedge on the skin dose was measured by inserting the motorized wedge (located at 18.6 cm from the source) in the beam, and measuring the dose from zero to a 2.0 cm depth, for all the above-mentioned fields. The collimator setting at 100 cm SSD defined the field sizes. The procedure described above was repeated to study the effect of SSD on skin dose. Three different SSDs were chosen for measurements (80, 100 and 120 cm). Twenty-five percent of the 20 × 20 cm^2^ field was blocked with Cerrobend block, with MLC, and the effect of the block on the skin dose at the three SSDs mentioned above was measured. The experimental setup is shown in Figure 1 and the diagram of the blocked field is shown in Figure 2.

## Results

**Figure 3:**
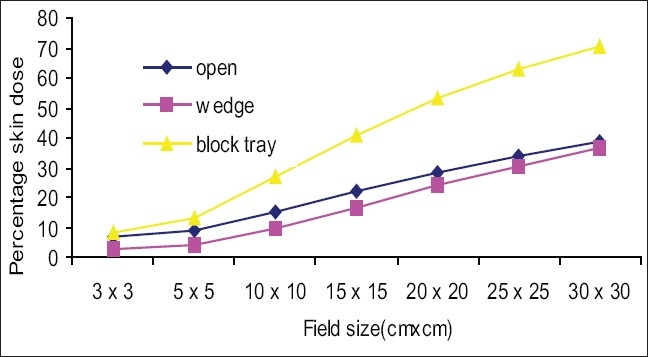
Comparison of percentage skin dose for open fields vs. motorized 60° wedge and acrylic block tray fields at 80 cm SSD

**Figure 4:**
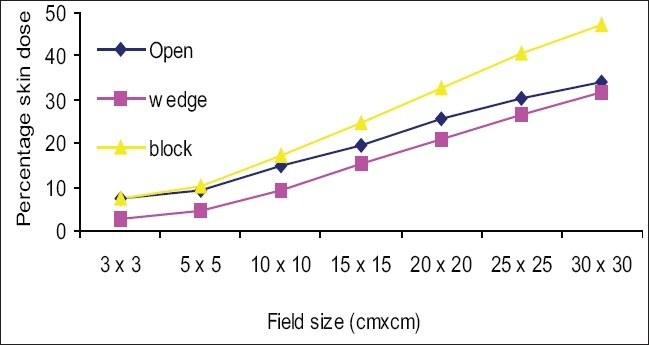
Comparison of percentage skin dose for open fields vs. motorized 60° wedge and acrylic block tray fields at 100 cm SSD

**Figure 5:**
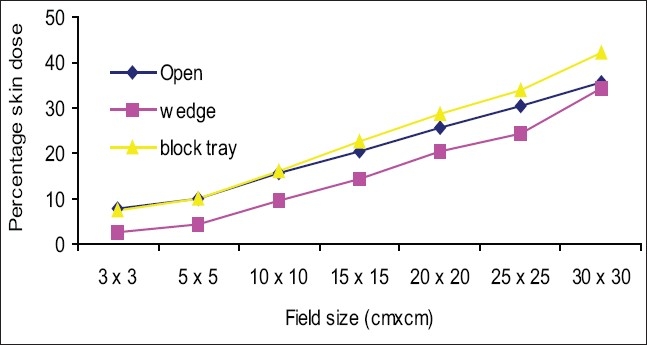
Comparison of percentage skin dose for open fields vs. motorized 60° wedge and acrylic block tray fields at 120 cm SSD

Figure 3 shows the percentage skin dose values for open, wedge, and block tray fields at 80 cm SSD. Skin dose increased as the field size increased. Skin dose values for wedge fields were lower than for open fields (for example 10 × 10 cm^2^ open and wedge field percentage skin doses were 15.5% and 9.9%). Skin dose values for tray fields were greater than for open fields and deviations were more in larger fields (for example, 10 × 10 cm^2^ open and tray field skin doses were 15.5% and 27%,and 30 × 30 cm^2^ open and tray field skin doses were 38.3% and 70.4%). Figure 4 shows the percentage skin dose values for open, wedge, and block tray fields at 100 cm SSD. Skin dose values for wedge fields were lower than for open fields (for example, the percentage skin dose values for a 10 × 10 cm^2^ open and wedge field were 14.8% and 9.5%). Percentage skin dose values for tray fields were greater than for open fields, and deviations were more for larger fields (for example, 10 × 10 cm^2^ open and tray field skin dose values were 14.8% and 17.2% and 30 × 30 cm^2^ open and tray field skin doses were 34% and 47.3%). Figure 5 shows the percentage of skin dose values for open, wedge, and tray fields at 120 cm SSD. Skin dose values for wedge fields were lower than for open fields (for example, 10 × 10 cm^2^ open and wedge field skin doses were 15.5% and 9.5%). Skin dose values for tray fields were more than for open fields and deviation was more in larger fields (for example, 10 × 10 cm^2^ open and tray field skin doses were 15.5% and 16.1% and 30 × 30 cm^2^ open and tray field skin doses were 35.7% and 42.1%).

Figures 3–5 show that skin dose values for 60° motorized wedge were less than for open fields at 80,100, and 120 cm SSDs. The motorized wedge eliminates secondary electrons, but generates new electrons. It may concluded that the number of electrons produced by the wedge were lower than the number of electrons eliminated by the wedge. The skin dose values for acrylic block tray fields were more than for open fields at 80,100 and120 cm SSDs.It may concluded that the effects of the blocking tray were quite significant and increased with an increased field size.

**Figure 6:**
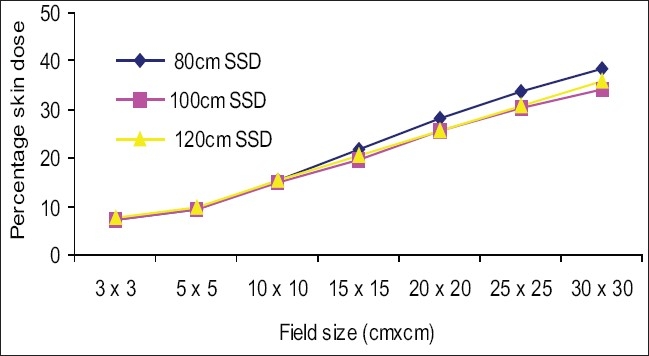
Comparsion of percentage skin dose at 100 cm SSD vs. 80 cm and 120 cm SSD for open fields

**Figure 7:**
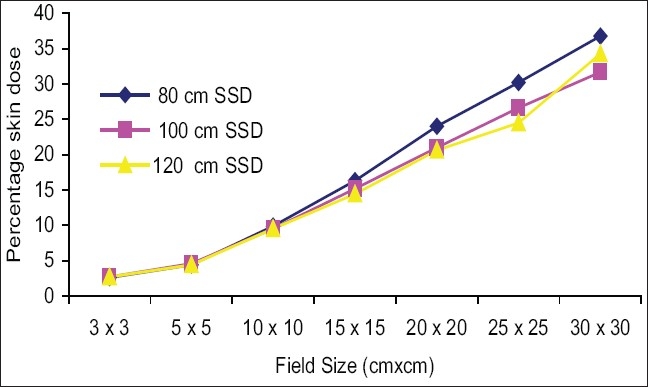
Comparision of percentage skin dose at 100 cm SSD vs. 80 cm and 120 cm SSD for motorized 60° wedge fields

**Figure 8:**
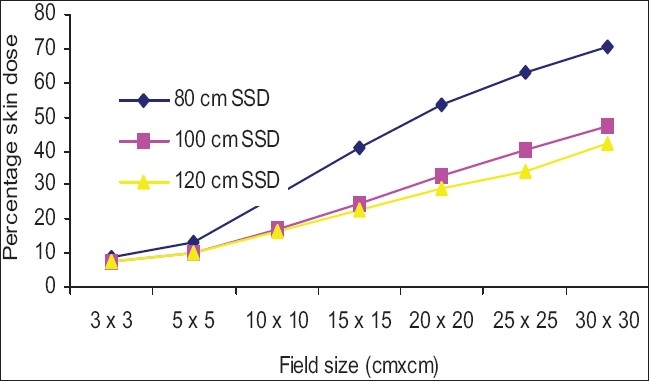
Comparison of percentage skin dose at 100 cm SSD vs. 80 cm and 120 cm SSD for acrylic block tray fields

Figure 6 shows the impact of SSD on the skin doses, for open fields. Skin doses at 120 cm SSD were slightly greater than at 100 cm SSD for all the measured fields, but deviations were not significant (deviation was less than 1% up to 25 × 25 cm^2^ field). Skin doses at 80 cm SSD were greater than at 100 cm SSD for all measured fields except 3 × 3 cm^2^ and 5 × 5 cm^2^ fields and maximum deviation was 4.2% for a 30 × 30 cm^2^ field. Figure 7 shows that percentage skin dose values at 100 cm SSD were nearly the same at 120 cm SSD up to a 20 × 20 cm^2^ field (deviations were less than 1%), and maximum deviation on the skin dose was 2.6% for a 30 × 30 cm^2^ field. Percentage skin dose values at 80 cm SSD were greater than at 100 cm SSD for all measured fields, except 3 × 3 cm^2^ and 5 × 5 cm^2^ fields and maximum deviation was 5.1% for a 30 × 30 cm^2^ field. Figure 8 shows that percentage skin dose values for block tray fields at 120 cm SSD were lower than at 100 cm SSD for all measured fields except 3 × 3 cm^2^ field and maximum deviation was 5.2% for a 30 × 30 cm^2^ field. Percentage skin dose values at 80 cm SSD were greater than at 100 cm SSD for all measured fields and maximum percentage skin dose deviation was 23.1% for a 30 × 30 cm^2^ field size. It may be concluded that the effects of the blocking tray on skin doses at low SSD were much more significant, and increased with increased field size.

**Figure 9:**
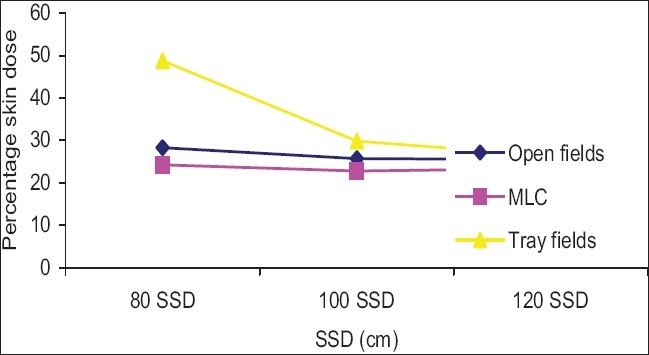
Comparison of percentage skin dose 20 × 20 cm2 open field vs. 25% blocked field with tray and MLC for various SSDs

Skin dose differences caused by using 25% blocks with MLC and cerrobend block in the 20 × 20 cm^2^ field at 80, 100, and 120 cm SSDs are given in Figure 9. Skin dose value for an MLC blocked field was lower than for an open field at the above-mentioned SSDs, but for the cerrobend blocked field, the skin dose was higher than for the open field. In the MLC blocked field, the irradiation field became smaller and the scattering decreased, but the cerrobend blocked irradiation field became smaller, however, secondary electrons produced from the blocking tray and blocks were more, hence the skin dose was increased.

**Figure 10:**
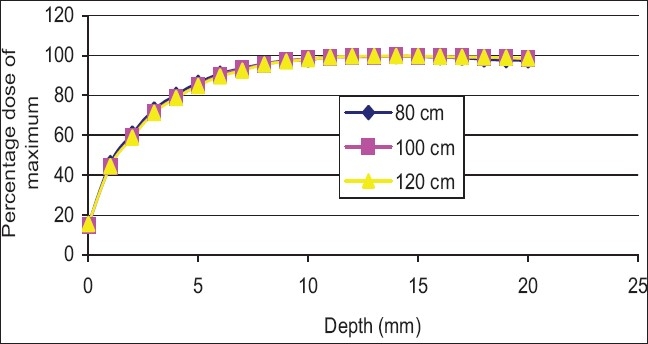
Percentage dose buildup curves for 6 MV at various source-tosurface distance for 10 × 10 cm2 open field

**Figure 11:**
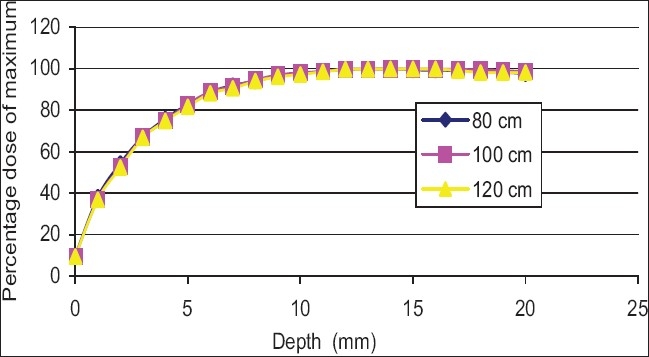
Percentage dose buildup curves for 6 MV photon at various source-to-surface distance for 10 × 10 cm2 wedge fields

**Figure 12:**
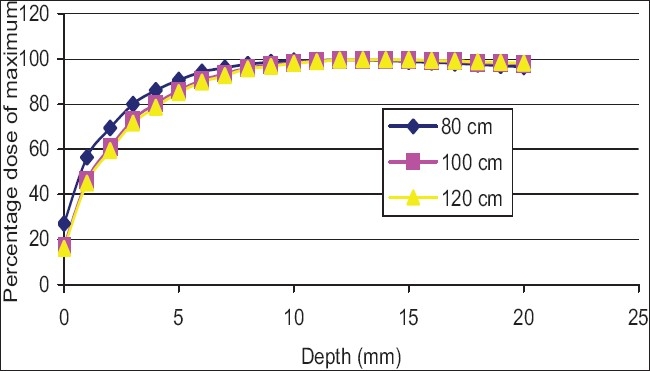
Percentage dose buildup curves for 6 MV photons at various source-to-surface distance for 10 × 10 cm2 field with block tray

Figure 10 shows the buildup curves for a 10 × 10 cm^2^ field (as at 100 cm SSD) at various SSDs from 80 to 120 cm for an open field. As can be seen the percentage dose, compared to the maximum, does not vary significantly with SSD. Figure 11 shows the buildup curves for a 10 × 10 cm^2^ field (as at 100 cm SSD) at various SSDs from 80 to 120 cm for a motorized wedge. No significant variation in buildup dose has been recorded over the range of 80 to 120 cm SSD. Figure 12 shows buildup dose curves for a 10 × 10 cm^2^ field size with 1.0 cm perspex blocking tray at various SSDs. A significant variation in the buildup dose was recorded over the range of 80 to 120 cm SSD.

## Discussion

Mega voltage X-rays are used for the treatment of deep-seated tumors, due to its skin sparing effect. This effect may be reduced because of contaminant electrons, which are generated outside the patient in the air or collimator.[12] There is a strong relation between the field size and skin dose. The skin dose increases as the field size increases. This increase is due to increased electron emission from the collimator and air. Skin dose values for open fields at various SSDs are shown in Figure 6. Percentage skin dose values at 120 cm SSD are slightly greater than those at 100 cm SSD, and maximum deviation is 1.7% for a 30 × 30 cm^2^ field size. This is probably due to the unique distal “x-ray” flattening filter for 6 MV.[16] Percentage skin dose deviations at 80 cm SSD are nearly the same up to 5 × 5 cm^2^ field sizes, and maximum deviation is 4.2% for a 30 × 30 cm^2^ field size. These results agree with the literature (for example the maximum percentage skin dose deviation measured by Batson *et al*,[14] was 4.0% for SSDs from 80 cm to 120 cm).

The skin dose values for acrylic block tray fields were higher than for the open fields. This effect was dominant for larger field sizes and at lower SSD. Figure 4 shows that skin doses for open and acrylic block tray were nearly the same for a 3 × 3 cm^2^ field, but it changed from 34.0% to 47.3% on adding acrylic block tray to a 30 × 30 cm^2^ field at 100 cm SSD. Figure 3 shows that the change in skin doses are more significant at reduced 80 cm SSD (for example 38.3% for an open 30 × 30 cm^2^ field and 70.4% for the acrylic block tray field). Percentage skin dose increases in the presence of acrylic block tray from 14.8% to 17.2% and 19.8% to 24.8% for 10 × 10 cm^2^ and 15× 15 cm^2^ fields at 100 cm SSD, respectively. These results agree with those in the literature (for example Tannous *et al*,[11] found 16% and 24% skin dose value in the presence of block tray 0.6 cm thick perspex for 10 × 10 cm^2^ and 15 × 15 cm^2^ fields, respectively). The block tray eliminates electrons from the upstream and generates new secondary electrons by itself.[2] The production of secondary electrons from the acrylic block tray is more than eliminated by the tray, and therefore, skin doses are increased. Figure 8 shows the effect of SSDs on the skin dose for various field sizes in the presence of a 1.0 cm thick acrylic block tray. Significant variations in skin doses were recorded over the range of 80 cm to 120 cm SSD. Maximum percentage skin dose deviation was 23.1% for a 30 × 30 cm^2^ field size. These results agree with the literature (for example maximum percentage skin dose deviation measured by Butson *et al,[*14] was 22% for a 40 × 40 cm^2^ field size with a 0.6 cm perspex block tray).

The skin dose values for the motorized 60° wedge fields increased as field sizes increased and were lower than those of the open fields [Figures 3–5]. It showed that the percentage skin dose difference between open and motorized 60° wedge fields were more (nearly 5.0% up to 20 × 20 cm^2^ fields) and less in large fields at 80,100 and 120 cm SSDs. Kim *et al*.[2] reported that a physical wedge (PW) both eliminated electrons from the upstream and generated electrons by itself. They noted that the number of electrons produced in a wedge was less than the number of electrons eliminated by the wedge, for smaller field sizes. According to their report, this effect was reversed only with larger field sizes and larger wedge angles. The measured skin doses for a motorized 60° wedge were 9.5% for 10 × 10 cm^2^ field sizes. The result agreed with that in the literature. For example, the skin dose value measured by Kim *et al*,[2] was 9.0% for 8 MV for 30° (PW) and 10 × 10 cm^2^ field sizes. According to Li *et al*.[7] the skin dose for a 30° (PW) field wedge was 10.4%. There was no significant effect of SSD on the skin doses up to 15 × 15 cm^2^ field sizes, but for larger fields skin doses at 80 cm, the SSDs were more than 100 and 120 cm and the maximum deviation was 5.1%.

The skin dose for a 20 × 20 cm^2^ open field was 25.7% for a 6 MV photon at 100 cm SSD (Butson *et al*,[15] measured 26.4% skin dose values for 20 × 20 cm^2^ open field sizes for a 6 MV photon at 100 cm SSD), and for 25% block of 20 × 20 cm^2^ field with acrylic block tray and MLC, it was 29.8% and 22.9% at 100 cm SSD, respectively. It was seen that the MLC block field surface dose was lower than that for the open field, because the irradiation field was reduced, but for the acrylic block tray field it was more than that for the open field, as the block irradiation field was reduced, but the secondary electron generated by the tray and block was more. It is seen from Figure 9 that the surface dose difference between the blocked field and open field decreased as the SSD increased, because as the SSD increased the number of electrons that reached the surface of the phantom (patient skin) decreased.

Figures 10 and 11 show no significant variation in the buildup dose, which was recorded over the range of 80 to 120 cm SSD. The field size was still quoted at the isocenter (i.e., the collimator positions remained unchanged), and this would explain the closeness of the measured buildup dose. The area inside the treatment head of the accelerator, which produced and allowed electron contamination to escape, remained constant as the SSD was varied. The electrons produced within the head of the accelerator were relatively high energy (i.e., the range of an electron up to 15 mm in water). When these electrons were required to travel say 20 cm, more or less, in air, it would not significantly change their range in the phantom by a sizeable amount. A similar scenario was expected for photons, which were produced in the collimator. Figure 12 shows the buildup dose for a 10 × 10 cm^2^ field size, with a 1.0 cm thick acrylic block tray, at various SSDs. A significant variation in the buildup dose was recorded over the range of 80 to 120 cm SSD. The clinical significance of these results was that for open and wedge fields, there was no significant change in the dose that was delivered to the skin and subcutaneous tissue with isocentric or extended treatment, however, with the use of blocking trays, the effect of the SSD changed the dose delivered to this region. An increase in skin dose could cause early radiation effects such as erythema or late radiation-induced effects such as hypoxia and relangiecresia

## Conclusion

The effects of source-to-surface distance produce minimal effects on the skin dose for open and wedge field beams, but significant effects are seen for block trays, for 6 MV x-ray energy. Skin dose values increase with decreasing SSD for fields with a blocking tray, due to the influence of electron contamination produced by the blocking tray. The use of a multileaf collimator for blocking removes the extra skin dose caused by the acrylic tray with decreasing SSD.
